# The importance of existential dimensions in the context of the presence of older patients at team meetings—In the light of Heidegger and Merleau-Ponty's philosophy

**DOI:** 10.3402/qhw.v10.26590

**Published:** 2015-02-19

**Authors:** Elisabeth Lindberg, Margaretha Ekebergh, Eva Persson, Ulrica Hörberg

**Affiliations:** 1 Academy of Care, Working Life and Social Welfare, University of Borås, Borås, Sweden; 2 School of Health and Caring Sciences, Linnæus University, Växjö, Sweden; 3 Board of Health Sciences, Faculty of Medicine, Lund University, Lund, Sweden

**Keywords:** Care, older patients, Heidegger, Merleau-Ponty, qualitative research

## Abstract

The aim of the present study is to explore interpersonal dimensions of the presence of older patients at team meetings. The theoretical foundation of the study is grounded in caring science and lifeworld phenomenology. The results from two empirical studies, that indicated the need for a more in-depth examination of the interpersonal relationships when an older patient is present at a team meeting, were further explicated by philosophical examination in the light of Heidegger and Merleau-Ponty's philosophy. The empirical studies were performed in a hospital ward for older people, where the traditional rounds had been replaced by a team meeting, to which the patients were invited. The analysis of the general structure and philosophical examination followed the principles of reflective lifeworld research. The philosophical examination is presented in four meaning structures: mood as a force in existence; to exist in a world with others; loneliness in the presence of others; and the lived body as extending. In conclusion, professionals must consider patients’ existential issues in the way they are expressed by the patients. Existence extends beyond the present situation. Accordingly, the team meeting must be seen in a larger context, including the patients’ life as a whole, as well as the ontological and epistemological foundations on which healthcare is based.

The phenomenon of the present study is the presence of older patients at a team meeting in a ward for older patients. The intention of the study is to gain a greater understanding of the findings from two empirical studies (Lindberg, Hörberg, Persson & Ekebergh, 2013a, 2013b). These indicated the need for further examination of the interpersonal relationships when an older patient is present at a team meeting. The present study is motivated by the need to explore dimensions of vulnerability and meaning, and how these dimensions can find their way into the tradition-bound situation of a team meeting. In an attempt to look beyond what is most evident and to gain a greater insight, the study has been conducted by creating a general structure and philosophically examining it in the light of Heidegger and Merleau-Ponty's philosophies. The empirical as well as theoretical foundations are presented below and a further clarification of the study is presented in Figure 1.

**Figure 1:**
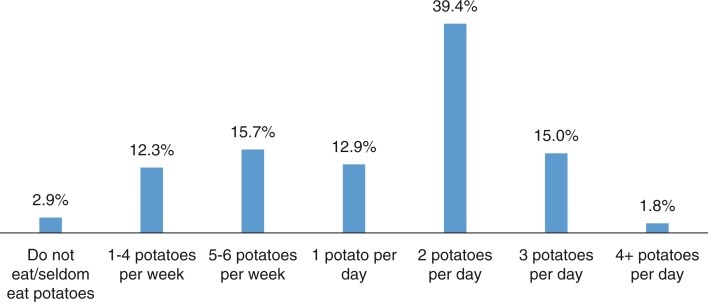
The research process.

## Empirical foundation

The empirical studies that form the basis of the present text were carried out in a ward for people >75 years of age. The ward had 15 beds, all patients came from their own homes, and the planned hospitalization was not to exceed 3 days. The patients who were treated in the department had, however, several medical diagnoses, and fatigue and anxiety were also common. Ultimately, most of the patients had a great need for care.

Patient participation is perceived as a right in many Western societies (Eldh, Ekman, & Ehnfors, 2006) and to increase the patient's involvement in their care, health care professionals initiated a project in which the older patient was invited to participate in a team meeting. The team meeting was, in this context, a way to develop the traditional ward rounds, and was held every weekday in the form of a “seated meeting.” Research indicates that the patients’ role in ward rounds is limited (Manias & Street, 2001; Molony, Horgan, & Graham, 2013; Sweet & Wilson, 2011; Swenne & Skytt, 2013; Weber, Stöckli, Nübling, & Langewitz, 2007) and questions relating to existential issues are often not recognized (Sweet & Wilson, 2011). To gain an understanding of how the patient's perspective was influenced by the change from a ward round to a team meeting, two lifeworld phenomenological studies were conducted.

Fifteen patients (three men and twelve women, aged 75–95), who had participated in a team meeting, were interviewed in the first study. The aim was to describe the caring, as experienced by the older patients in a ward for older persons, with a specific focus on the team meeting (Lindberg et al., 2013a). Nine nurses, who had experienced team meetings in which patients participated, were interviewed in the second study. The aim of the study was to highlight the experiences of nurses of the participation of the older patients in team meetings (Lindberg et al., 2013b). The results from these studies showed that the team meeting included existential, emotional, and relational dimensions and the results raised new questions related to the gaining of a greater understanding of interpersonal relationships and existential dimensions when an older patient is present at a team meeting.

## Theoretical foundation

In the present study, a phenomenological lifeworld perspective creates the foundation for caring science as well as for the research approach. The study approach is reflective lifeworld research (RLR), as described by Dahlberg, Dahlberg and Nyström (2008). RLR is based on the phenomenological philosophies of Husserl (1970/1936, 1977/1929) and Merleau-Ponty (1968/1964, 2011/1945). The goal of RLR is to describe human lived experience to achieve a new or better understanding of a phenomenon of interest. In the present study, this translates to the presence of older patients at a team meeting in a ward for older patients.

In a caring science context, the lifeworld perspective has been shown as constituting a possibility for developing a holistic care. A caring science based on a lifeworld perspective takes into account humans as a whole, while simultaneously affirming the ambiguity of existence (Dahlberg, Todres, & Galvin, 2009). Human beings exist side by side with other humans and in a lifeworld perspective this relational aspect has been described by Merleau-Ponty (2011/1945) as intersubjectivity. In the encounter between health care professionals and patients, intersubjectivity can be a powerful source in the search for health, but there is always a risk that the intersubjective dimensions can cause harm. People can, for example, feel violated and neglected, when their needs are ignored (Todres, Galvin, & Dahlberg, 2007). Caring demands responsiveness to the lifeworld of the other, which means to listen, to touch, and be touched without avoiding the ambiguity of existence (Dahlberg et al., 2009).

As previously described, the context for the study is the team meeting. The aim of these meetings varies, but the common denominator is that they constitute an arena for discussions about care. According to earlier research, an encounter with the intention to be caring must be based on mutuality (Gustafsson, Snellman, & Gustafsson, 2012; Holopainen, Kasén, & Nyström, 2012) and the carers need to have courage and strength to stand by the patient's side in moments of suffering (Holopainen et al., 2012; Snellman, Gustafsson, & Gustafsson, 2012). To encounter entails being in a space of where humans are not separated from each other and are in a mutual existence (Holopainen et al., 2012). The nurse–patient interaction can also be a resource for hope (Haugan, Moksnes, & Espnes, 2013), whereas increased suffering for the patient can be the outcome when dimensions in relation to existence are ignored or not met (Arman, Rehnsfeldt, Lindholm, Hamrin, & Eriksson, 2004; Galvin & Todres, 2009, 2013; Lindholm & Eriksson, 1993).

In summary, caring science and lifeworld phenomenology contribute to highlighting the needs for the development of understanding of existential and interpersonal dimensions of caring encounters. This together with research and the results from our empirical studies (Lindberg et al., 2013a, 2013b) raised new questions and called for further examination of experiences in relation to the presence of older patients at a team meeting. As the context of the studies are related to a ward for older people, the existential dimensions as well as the interpersonal relationships are seen against a background of increased vulnerability of human beings as time passes by. The aim of the present study is thus, to explore the interpersonal dimensions of the presence of older patients at team meetings.

## Method

The present study was conducted in two steps. First the essences from the empirical studies were analysed, which resulted in the general structure. Second a philosophical examination of the general structure was conducted to further examine the understanding of the phenomenon.

The creation of a general structure has previously been described by Dahlberg et al. (2008) and Hörberg (2008). A general structure means that the essences of two (or more) results are integrated in a new analysis and can be seen as a synthesis and abstraction of the results of the included studies. The analysis included a movement between the whole and the parts. A new whole emerges when patterns of meaning from the two empirical studies are intertwined. The analysis process began with open readings of these essence descriptions, which were merged together to create a new foundation. Guided by the research questions, further development of the understanding of what constituted a patient's presence at a team meeting was possible. Examples of new research questions were viewed against the background of a new meaning structure: How does the presence of older patients at the team meeting manifest itself? How can the patient's presence at the team meeting be understood at a deeper level? What does the patient's presence mean for the aspects of interpersonal relations during the team meeting?

As described by Dahlberg et al. (2008), the general structure is to achieve a high level of abstraction. Insofar as it has been possible, this has been sought in the writing of the general structure. However, as the general structure is based on the synthesis of two perspectives (the patient and the nurse), there has, in some cases, been a need to clarify who the subject is, something that may diverge what is common practice in abstract description (essences) in RLR. Based on the variation between the parts and whole, and with support from the phenomenon and research questions, a general structure was formulated.

To gain a greater understanding of the phenomenon, a philosophical examination of the general structure was carried out. Philosophical texts by Heidegger (1962/1927) and Merleau-Ponty (1968/1964, 2011/1945) were used to further the understanding of the current phenomenon. Heidegger contributes, through his texts, to a greater understanding of the existence, in which man is “thrown” into a world with other humans, and where human vulnerability in a finite (spatial/temporal) existence is ever present. In this, to a large extent unpredictable, existence, Heidegger also means that the future holds opportunities. Merleau-Ponty contributes to the understanding of intersubjectivity in the interpersonal encounter and furthers our understanding of the body's central place in existence. Heidegger and Merleau-Ponty's texts are written in a different time and context than those of a team meeting in a ward in Sweden today. Nevertheless, there is a valuable insight into the existential message of the texts, which can provide a greater understanding of the complexity of human beings and interpersonal relationships. The intention has been to read and apply the philosophical texts with great humbleness, and in such a way as to give strength to and further the understanding of the empirical data, rather than interpreting and analysing the texts from a philosophical perspective.

The philosophical examination was carried out in such a way that selected parts from the general structure were problematized against parts of the philosophical texts. The encounter between the general structure and the philosophical texts generates a dynamic process, which must be open and reflected upon as long as possible. In accordance with RLR (Dahlberg et al., 2008), the general structure and the philosophical texts, alternately, have appeared as a figure and background. For example, by placing the general structure as a figure against a background of the philosophical texts, the importance of gaining a greater understanding of the imbalance in the situation emerged. By placing the philosophical texts as the figure and the general structure as the background, the search could be directed to more specific parts. For example, the reading of Merleau-Ponty (1968/1964, 2011/1945) contributed to an in-depth understanding of how intersubjectivity becomes relevant when the older patient attends a team meeting. In this process, a new understanding has emerged resulting in four “meaning structures” (in the absence of methodological literature in the field, the term “meaning structure” was chosen to highlight both the intention of a search for meaning as well as the need for a structure of the presentation).

As with any analysis with a RLR approach, openness, adherence, and bridling of the understanding permeated the analysis of the general structure as well as the philosophical examination (Dahlberg et al., 2008). However, there is a risk that the openness, adherence, and bridling is influenced because the material is well known from the past; on the contrary, there is also the need to further the understanding of the previous studies that form the bases to move forward with the material. Openness here means developing the understanding of the phenomenon by letting the essences from the empirical studies merge together into a new whole. Adherence is located in the nature of the present study, as it has emerged from the need to gain a greater understanding of the earlier studies. Bridling is of central importance, and as Dahlberg et al. (2008) describes, bridling does not mean putting aside preconceptions, but to keep the developing understanding under control to give justice to the phenomenon, letting new meanings emerge. Reflection on the meaning of that which shows itself is of importance, and reflections in the supervisory group and seminars have been supportive in this process.

## Result

The results will be presented, beginning with a description of the general structure, followed by the philosophical examination, which is presented in four meaning structures: mood as a force in existence; to exist in a world with others; loneliness in the presence of others; and the lived body as extending.

### General structure

The situation in which the team meeting occurs is characterized by an imbalance between the participants. In this imbalance, it is obvious who is in possession of the power and who is subordinate. Attendance at the team meeting is taken for granted by the professionals, whereas an invitation is needed for the patient; accepting the invitation means stepping into the unknown. Vulnerability and a sense of being lost, already present in their existence (as a result of dependence, ageing, and disease), is thus likely to be reinforced. The vulnerability is balanced against the possibility of being part of the care and being part of a unit.

The atmosphere in the room is affected by the presence of the unknown (the patient). Emotions and expectations affect the atmosphere and disturb an otherwise secure structure. The presence of the patient can enable a mutual commitment and a meeting that is experienced as meaningful, but the situation can also be characterized by a feeling of disorientation among the participants.

A prerequisite for participation is a common expression, in which dialogues as well as interpersonal relationships are essential. The absence of a common expression leads to a growing distance between the participants, and the tension can fall like a “curtain,” covering the situation. Paradoxically, participation and invitation can turn into loneliness and give rise to feelings of abandonment, and feelings of being neglected and invisible can also occur. Going beyond familiar borders, as well as working to create conditions for participation for everyone present, involves the risk of being excluded from the unity.

The time is essential in relation to the patient's presence at the team meeting. The team meeting is not limited to “clock time” or to the place as a room. The team meeting is, instead, expanded and interwoven in the care and in life as a whole. The participants are expected to appear and remain in the room during the given period of time. It takes courage and strength to observe the time as lived, because it will challenge the known structure as well as claim the clock time. A caring that is experienced as meaningful can be created in the present moment, but may as well be created over a prolonged period of time. The essential is to acknowledge the whole human being, listen, and take care of his/her life history and, based on this, co-create a viable path into the future.

### Philosophical examination

#### Mood as a force in existence

In a strict organizational sense, the team meeting is an opportunity for planning the care and for planning the patient's discharge from the hospital. The general structure, however, indicates that this description is not sufficient; the team meeting is something more, something deeper, and something that touches and sometimes shakes up the participants. Moods, emotions, and relationships seem to take great presence in the interactions between the participants. Heidegger (1962/1927) describes that in life we are always together with others, but we are not only with others. Heidegger also goes a step further, suggesting that “the self” is created in the company of others. As we shall see in the following text, the team meeting is an opportunity where people can acknowledge and support each other, but it is also a situation where people can walk past each other, objectify, and omit. The meeting between the various actors (professionals and patients) is a movement between security and disorientation. The patient's presence in the room is unfamiliar for all participants, and adds an extra dimension of immediacy (by way of, for example, emotions) that requires attention to meet and receive. For the patient, the team meeting can be an emotional situation, discussions about home situations and illness confront the patient with their own vulnerability, and tears cannot always be held back. When everyday life no longer works and the need to accept help from others occurs, this is no longer only an issue demanding practical solutions. To go from independence to dependence may mean something more dramatic, and also arouse feelings of shame.

In Heidegger's philosophy, mood is something that is always present; man is “tuned” in its existence. Unlike emotions, which are more related to events and thoughts, the mood is already present, and Heidegger (1962/1927, p. 176) writes: “A mood assails us. It comes neither from ‘outside’ nor from ‘inside’, but arises out of Being-in-the-world, as a way of such Being.” The persons involved in the team are in a certain mood when entering the situation and simultaneously the current situation also creates a mood. The mood is, in Heidegger's philosophy, connected to our “thrownness” in existence. To be “thrown” into existence includes the possibility and inevitability of one's own mortality. The need for human beings (Dasein) is to assume these possibilities, that is, the need to be responsible for one's own existence. In the patients’ descriptions, the mood is present as a force in existence that both drains and strengthens the will to live. The mood contributes to a closeness of emotions. In dark moments, loneliness, vulnerability, and the finitude of life paralyze, and in other moments, joy and gratitude create happiness and a will to live. In the often formal structure of the team meeting, this proximity to emotions contributes to both a sense of loss over how to handle the emotions, as well as to a feeling of warmth and thoughtfulness in the situation.

When entering the situation, the professionals also are in a mood, but whereas the patient's mood is linked with their life situation, the professionals’ mood is in relation to their professional mission. There is a longing to perform well and a serious longing for confirmation. The confirmation may be about being recognized by colleagues, but there is also a longing for a feeling within oneself related to having done a valuable job for the patient. The constant lack of time and the fragmentation of the profession often lead to a mood characterized by inadequacy. In this mix of moods, in which the participants find themselves during the team meeting, rational, sometimes life-changing decisions are expected to be made in an often very limited period of time.

The mood can thus be said to influence the caring encounter. The patients sense the professionals’ stress and can in some situations choose to avoid asking for help and support in an effort to protect the personnel from further pressure. In turn, the professionals can choose to acknowledge and confirm the mood of the patient, and thus create a caring encounter, or they can choose to neglect moods expressed by the patients, and thus contribute to an encounter at worst described as non-caring.

#### To exist in a world with others

At the team meeting, the encounter between the participants requires some form of understanding of the other's situation. The question is how an understanding of other humans can be developed. Through our shared experience of being humans, we can develop the ability to empathize with each other's vulnerability. Further development of this understanding can be obtained through the texts of Merleau-Ponty (2011/1945), who describes intersubjectivity as the possibility to approach the other's experience. We are different from each other in the same way in which we belong together. Through our own experiences as thinking, feeling, and acting humans, we can develop the idea that other humans are thinking and feeling, and that their actions have a purpose and an objective. We can experience that another human feels happiness, grief, or anger, but we cannot feel how this feeling is experienced by another. We can share experiences with other humans, but we do not experience in the same way. A possibility for developing caring can be found in the tension between our inkling of the others emotions and the others concrete experiences. Intersubjectivity is not just about how a lived body understands another lived body. In the space between two lived bodies, something more, something in common, is created.

The patient's presence at the team meeting opens up the possibility of a shared vision, in which the professional knowledge and the patient's lived experience can, to use Merleau-Ponty's (1968/1964, p. 142) words, “invade” each other. During the team meeting, there is the need for a variety of perspectives on how best to provide support to the patient. The different perspectives contribute to create, as much as possible, a picture of the patient's whole situation, and these different perspectives can contribute to co-create understanding.

Humans can thus not be seen as distinct entities, separated from other entities without influence on each other. Instead, Merleau-Ponty describes humans as extending towards the world, and in this way, in a broader sense, becoming an intertwined part of the world. In extending towards the world, humans affect and are affected by other humans. Those who participate in the team meeting are not just individuals, separated from one another and “placed” in the room for a specific mission. There is also something more between the humans in the room, something created by and arising out of the unique encounter. Maybe one of the patients puts this “something” into words when he described that he felt “enveloped” by the situation. For this patient to feel enveloped was an expression of security and affirmation, and the patient dared to surrender to, not only the other persons in the room, but also to the situation, which was created in the room. To feel enveloped can then be seen as something that opens up, towards events and relationships. But enveloping can also be understood as something that comes too close in a way that it almost becomes suffocating, threatening the integrity; some situations require distances to be endured. An example of such a situation is when vulnerability is too clearly exposed, without sensitivity for the patient's reactions, or when practical solutions are presented without giving the patient the possibility to reflect. Unless the patient has time and opportunity to be involved in the situation, which in all kindness is meant to support and help, it can easily be transformed into a force that reinforces the patient's vulnerability.

#### Loneliness in the presence of others

During the team meeting, situations arise in which the participants’ vulnerability becomes obvious. Through interest and curiosity for the other, possible tensions can be overcome, but by maintaining locked positions and by a lack of knowledge, the professionals may also give themselves a mandate to an interpretative privilege of the others’ experiences. Whether the patient becomes an object or subject in the current situation is mainly determined by the professionals’ attitudes, and ability to reflect on what it is like for the patient to be in this situation. This is exemplified in a situation when a patient is brought to the team meeting, despite the fact that he/she is very ill and confused. The patient becomes exposed by the professionals’ gazes, who rapidly lose interest in the confused and dazed man/woman, and instead began to talk among themselves, discussing his/her care in an objectifying way.

It is revealed in the general structure how the asymmetrical power structure of the team meeting is likely to reinforce the patient's vulnerability and sense of homelessness and loneliness. It also reveals how important the professionals’ attitudes are. By providing support and confirming the patient, the team meeting can become a place for care, but when uncertainty about the purpose of the patient's presence in the room takes over, the meeting may become “non-caring.” The situation described above, when the patient is brought to the team meeting despite his/her vulnerability, is one in which the patient is excluded and where the focus is on the professionals’ objective assessment of the patient. The opportunity offered by intersubjectivity to imagine the other's experience (and in the current situation the patient's vulnerability) is not present in the described situation. The patient is present in the room but excluded and homeless as a subject.

The situation described above exemplifies loneliness. Heidegger (1962/1927, p. 157) argues that loneliness is a form of, what in Heidegger's philosophy is termed as “Being-with”; “The Other can be missing only in and for a Being-with.” The ambiguity of existence emerges in the variations of loneliness and “Being-with” in which humans find themselves in. As humans, we are, at the same time, completely alone and in constant relation to others. Ageing and disease restrict access to other humans and to the possibility of relationships. Loneliness becomes more present in life, and this presence can be accepted or challenged. Regardless of how loneliness is met, it may challenge the well-being, and must be examined when health and well-being are discussed. During the team meetings, the patients approach issues in relation to loneliness and how it affects well-being. With patients related to existential issues (for example, loneliness), the premise of the team meeting is put to the fore. If the approach to the team meeting is that it is an opportunity for action and problem solving, a tablet against anxiety may be the solution to the loneliness, but as mentioned earlier, the situation *per se* can offer an opportunity. To be seen, acknowledged, and listened to in the moment is important and can contribute to confidence and strength.

The imbalance described between the actors of the team meeting suggests that the nurse in various situations can be left alone in the caring. Loneliness occurs for the nurses, mainly in situations when they have the courage to go beyond established limits, and when they take up the fight for the patients against their colleagues. The patient's presence at the team meeting creates tension in the professional group. By keeping to a safe structure and not challenging old habits, contradictions in the professional group are avoided. In loneliness and perhaps also in fear of loneliness, a sense of being lost arises in the situation. The worthy idea concerning the patients’ participation and presence at the team meeting is challenged by the fear of being abandoned, which is present among both nurses and patients. There is something paradoxical that we are created in relation to each other, but at the same time, it is in relation to each other that we experience loneliness. Loneliness in the described situations is not a peaceful self-imposed loneliness, but bears the touch of a loneliness that needs to be challenged and that requires courage and strength to endure.

#### The lived body as extending

The lived body spreads out in various dimensions, such as in time, interpersonal relationships, and space. The team meeting does not arise out of nothing and by itself it does not take place as in an isolated vacuum. The “Being-with” (between the professionals and the patient) is not only created in the here and now of the team meeting but is also established during current hospitalization as well as through past experiences of caring and non-caring encounters. Events during previous care episodes persist and create insecurity and unease in the here and now situations.

One woman describes how she, after a previous care episode, received a letter. The message in the letter was that she was considered as being diagnosed with dementia. The woman describes how shocked and upset she became when receiving the letter; nobody had told her about the diagnosis during her stay in the hospital. Now that she is invited to participate in the team meeting, the thoughts of the previous situation came back to her and she describes how she prepared to “defend” herself if the same “accusation” came up again.

Merleau-Ponty (2011/1945) describes how the lived body is extending towards the world and creates conditions for interpersonal dimensions. The extension of the lived body makes a connectedness with other humans possible and makes it possible to influence other humans. Positive as well as negative relationships between humans are possible through the fact that humans’ worlds are shared and cohesive. The patient, who in the previous description received information about a diagnosis of dementia, describes that she felt annihilated and violated when receiving the letter. The letter's message of dementia reduced the woman to mere biology. When being invited to the team meeting, not just the feeling of being annihilated is brought back to her, she also brings the feeling with her into the situation, mobilizes strength to meet it if it occurs again, and she is getting prepared to “defend herself.” The previous situation and the feelings it gave rise to stretches out and infiltrates the new situation which the woman is about to enter. What happens here and now persists in humans and will be present as thoughts, feelings and, at worst, insecurity about how future situations will be perceived and understood.

In addition to the temporal dimension of the lived body as extending, there is also a dimension that can be described as intersubjective. This dimension can be exemplified in how well-being stretches out beyond the self and towards other significant people, animals, or interests. When the own body is letting you down, well-being and a zest for life can emerge from meaningful relationships. Freedom and the opportunities of existence exist in the major and minor events of everyday life, where the driving force can be a longing for the family, a pet, or the ability to be able to bring your own food from your own kitchen. Freedom and humans’ “existential potential-for-being” are linked to Heidegger's description of humans’ “thrownness” into existence; time and the spirit in which we live, as well as ageing and disease, mean a certain element of “throwness” into existence. We did not choose to be born into a certain time, and we did not choose to grow old or get ill. At the same time, according to Heidegger, “throwness” in some sense implies a future possibility. In this way, our lives include an ambiguity in which a non-voluntary temporality, including ageing and disease, is a part and, at the same time, the future as a possibility is there to seize. Part of the care can thus be that the carer in communion with the patient tries to catch sight of future possibilities in the situation.

Thus, the lived body is extending into a temporal as well as intersubjective dimension. The lived body is also extending into a spatial dimension. In the space between human bodies, opportunities to encounter and co-create, as well as a risk of loneliness and increased vulnerability exist. According to Merleau-Ponty (2011/1945), language constitutes a central medium through which we can share our own and others’ human experiences; “In the experience of dialogue, a common ground is constituted between me and another; my thought and his form a single fabric” (Merleau-Ponty, 2011/1945, p. 411). The dialogue constitutes a possibility to coexist, but in the general structure the lack of a common language emerges as an aggravating circumstance. An institutional framework, formal roles, and unfamiliarity in encountering existential issues contribute in various ways to the difficulty in finding a common language.

Gestures, as well as language, can contribute to opportunities and increased vulnerability. Merleau-Ponty (2011/1945) describes how other human gestures can be understood through an interaction between gestures and intentions: “Everything happens as if the other person's intention inhabited my body, or as if my intentions inhabited his body” (Merleau-Ponty, 2011/1945, p. 191). The positions of the bodies in the room can signal both proximity and distance. And because the patients can have difficulties in both hearing and seeing, the position in the room can complicate the patient's opportunity to participate in the conversation. One extreme is when the professionals position themselves facing a computer screen, with their backs to the patient, whereas the other extreme can be seen when the professionals embrace the patient. The patient's situation and life story take hold of the carers, and they respond by a gentle touch or by putting an arm around the patient. The worlds of the carers and the patients are brought together by the physical as well as existential touch.

### Methodological considerations

The attempt to use parts of Heidegger and Merleau-Ponty's philosophy is conducted with awareness of the fact that their texts include many more aspects than can be included in this study. Based on some parts of the philosophers’ works, the intention has been to contribute to a new understanding and to elucidate an extra dimension of the patient's presence at team meetings. There is always a risk when things are taken out of context that fragmentation can occur, which can lead to misunderstanding. To choose both Heidegger and Merleau-Ponty also represents a risk that could result in further fragmentation. The choice is motivated by the fact that they, in relation to the intersubjective aspects of the patient's presence in the team meeting, can complement each other. The philosophy as well as the application of the philosophy in the context of “older patient's presence at the team meeting” has been reflected and discussed in seminars and in the research group.

The intention of the philosophical examination is not to provide solutions for how the team meeting shall be conducted to realize patient participation. There is no simple answer for how the patient's presence at the team meeting improves care; instead, the philosophy sheds light on further questions. However, maybe it is through the questions that the situation can evolve. Hopefully, this study can contribute to raise awareness of dimensions that would otherwise have been hard to recognize. The philosophical texts have contributed to a greater understanding of human relations, and contributed to put into words the existential dimensions of the team meeting.

## Concluding reflections

This study highlights the importance of interpersonal relationships in a situation often characterized by formality and traditions. Although there is a framework with its integral aim for the team meeting, the situation is highly influenced by the people present, and the impact that humans have cannot be ignored. Human beings (Dasein) are never free from moods. When a mood is mastered, it is mastered into a counter mood. At the same time, the atmosphere in the situation *per se* creates a mood that at best can ease the burden, but at worst can add insult to the injury by, for example, exposing the participants to ignorance and unreflected attitudes. As humans, everyone attending brings with them thoughts and feelings into the situation, and thus contributes to fill the intersubjective space. In this weave of interpersonal relationships, the patients need support to be able to find their space and regain well-being and self-dependence. For the situation to be caring and meaningful for the patient, there needs to be convergence in terms of the purpose of the meeting, and an intention to consider the patient's situation from a holistic perspective. When the patient's unique need creates the foundation for the situation, the interpersonal relationships can also be understood as caring.

As explained in the philosophical examination, the professional's situation is demanding and complex. A longing for a genuine encounter with the patient is resisted by an organization under constant time pressure. In conclusion, both the patients’ and the professionals’ situations need to be considered on an existential level, as they both are at risk of loneliness as well as resignation: to be exposed to inauthentic care and to feel forced to deliver the latter affects humans, albeit differently.

The development of the team meeting towards a more caring perspective should not be seen as an attempt to eliminate the medical discussions that are traditionally performed in these contexts. The development should, instead, be seen as an attempt to integrate and expand the perspective on the patients’ situation. The human is both biology and existence interwoven into a complex whole, and it is this whole that needs space in situations where humans are at their most vulnerable.

In conclusion, the present study supports and develops previous research focusing on the importance of relational aspects of caring. The lifeworld perspective, which has a common root for both caring science and a research approach, clarifies the need for a reflected patient perspective in caring encounters.

Considering the situation in which the team meeting occurs as an occasion for care creates possibilities for seeing the patient's lifeworld, and hopefully, professionals working in a clinical context can take advantage from the result in various ways. For example, professionals need to be aware of the moods they bring in to the situation; they also need readiness to acknowledge moods expressed by the patient as well as readiness to touch existential issues. How the encounter is understood and interpreted will remain in the memory of the patient; for the professionals this involves a great responsibility, as the situation here and now will influence the patient in the future. Existence extends outside the room and the situation, which means that the team meeting must be seen in a larger context, including the patient's life as a whole as well as the ontological and epistemological foundations upon which healthcare is based.
